# Sleep restriction and age effects on waking alpha EEG activity in adolescents

**DOI:** 10.1093/sleepadvances/zpac015

**Published:** 2022-05-10

**Authors:** Ian G Campbell, Elizabeth I Kim, Nato Darchia, Irwin Feinberg

**Affiliations:** Department of Psychiatry, University of California Davis, Davis, CA, USA; Department of Psychiatry, University of California Davis, Davis, CA, USA; Ilia State University, Tbilisi, Georgia; Department of Psychiatry, University of California Davis, Davis, CA, USA

**Keywords:** adolescence, development, sleep loss, alpha

## Abstract

**Study Objectives:**

To understand how sleep need changes across adolescence our laboratory is carrying out a longitudinal dose–response study on the effects of sleep duration on daytime sleepiness and performance. This report focuses on the relation of the waking alpha (8–12 Hz) electroencephalogram (EEG) to prior sleep duration, whether this relation changes with age, and whether decreased waking alpha power is related to changes in daytime sleepiness, vigilance, and executive functioning.

**Methods:**

Study participants (*n* = 77) entered the study at ages ranging from 9.86 to 13.98 years and were studied annually for 3 years. Each year participants completed each of three time in bed (TIB) conditions (7, 8.5, or 10 h) for four consecutive nights. Waking EEG was recorded on the day following the fourth night.

**Results:**

TIB restriction and resultant sleep loss were associated with reduced alpha power with the effect being stronger for the eyes closed condition. TIB restriction altered the power spectrum within the alpha range by increasing the frequency of maximum alpha power. Alpha power decreased with age, but the effect of TIB restriction did not decrease with age. Reduced alpha power was associated with small but significant increases in subjective and objective sleepiness but was not associated with changes in vigilance or executive functioning.

**Conclusions:**

We interpret the alpha depression following sleep loss as incomplete sleep dependent recuperation that contributes to daytime sleepiness. The absence of a decrease in TIB effects with age indicates that this sleep need measure does not decrease over early to mid-adolescence.

**Statement of Significance**

This study systematically altered time in bed to test effects of sleep loss on waking EEG activity. Sleep loss depressed waking alpha power, and alpha power declined with age, but the sleep loss effect did not decrease with age. Reduced alpha power was associated with an increase in daytime sleepiness. As a practical matter, the decrease in alpha activity with sleep loss may point to an easily obtained and objectively quantifiable measure of sleep adequacy.

## Introduction

There is increasing public health concern that adolescents obtain insufficient sleep, impairing cognitive and social function [1, 2]. Our laboratory shares this concern but believes there are insufficient data on adolescent sleep need to provide firm recommendations for needed sleep durations across age. To obtain such data we have been carrying out longitudinal dose–response studies of sleep duration effects on daytime sleepiness and cognitive function in adolescents. We have found that prior sleep duration strongly affects one objective measure of daytime sleepiness, the multiple sleep latency test (MSLT). Data from the first 3 years of this study [3] showed that sleep latency on the MSLT increased with increasing prior time in bed (TIB) from 7 to 8.5 h and further increased when TIB was increased from 8.5 to 10 h. This reduction in objectively measured sleepiness with increasing TIB diminished with increasing age from 10 to 16 years, i.e., longer TIB produced a greater beneficial effect on sleep latency in younger adolescents.

Another objective measure of daytime sleepiness involves recordings of waking alpha EEG. Akerstedt and Gilbert [4] found that increasing sleepiness during a night of total sleep deprivation is accompanied by an increase in eyes-open alpha EEG power and a diminished response of waking alpha power with closing of the eyes. This observation suggested using the ratio of eyes closed alpha power to eyes open alpha power (alpha attenuation coefficient—AAC) as a marker that could be used to objectively measure sleepiness. Subsequent studies showed that a decrease in the AAC was correlated with decreasing sleep latencies on the Multiple Sleep Latency test [5] and higher ratings of sleepiness on the Karolinska Sleepiness Scale [6].

We recently showed that presumptively insufficient sleep durations reduced waking EEG power in a wide range of EEG frequencies [7]. In the current report, we focus on the effects on alpha EEG. Alpha waveform frequencies (8–12 Hz) are characteristic of the waking EEG in humans and have been intensively studied since the discovery of the EEG almost 100 years ago. They are most prominent in occipital recordings when subjects are relaxed with eyes closed. Both increases and decreases in arousal level reduce alpha EEG. Thus, alerting stimuli reduce alpha EEG activity, and the disappearance of alpha waveforms is one of the earliest EEG indicators of the transition from waking to sleep. We focus on alpha also because previous studies have demonstrated an association between alpha EEG rhythms and working memory and attention [8–10].

Here, we expand on our previous finding by not only testing the effects of reduced time in bed (and reduced sleep duration) on waking alpha EEG power but also the change in alpha with age. We propose that the response of alpha EEG to prior sleep duration is a measure of sleep need, and we evaluate whether this measure of sleep need changes with age across early and middle adolescence in this longitudinal study covering ages 10–16 years. Furthermore, we determine how TIB restriction and age affect the power spectrum within the alpha range, paying particular attention to effects on the frequency at which alpha power is at its maximum. Finally, we evaluate whether the changes in waking alpha power following four consecutive nights of TIB restriction are related to changes in objective and subjective daytime sleepiness, vigilance, and executive functioning.

## Methods

### Participants

Seventy-seven participants enrolled in and completed at least one year of this longitudinal study. Recruitment details have been previously published [11]. Seventy-six of these subjects participated in year 2 of the study, and 67 participated in year 3. The most common reason for withdrawing from the study was conflicts between school, work, or extracurricular activities and the required time in bed schedules of this study.

Parents provided informed consent and children over 12 years of age provided assent. During the consent interview participants were screened for the following exclusion criteria via an interview with a parent: diagnosed psychiatric or behavioral disorder, epilepsy, head injury resulting in loss of consciousness and symptoms lasting longer than 24 hours, diagnosed sleep disorder, a Sleep Disturbance Scale for Children *t*-score greater than 70, visual problems that could not be overcome with corrective lenses, manual dexterity problems that would interfere with performance tests, and current use of medication affecting the central nervous system. Most participants resided in the university town of Davis, CA. The sample of 77 participants is not ethnically diverse. Fifty-three participants were non-Latino white.

### Study design

Each year each participant completed three different TIB schedules with four consecutive nights with either 10, 8.5, or 7 h TIB. For all three experimental TIB schedules, three nights with 8.5 hours TIB preceded the four experimental nights. Subjects kept their habitual rise time and altered their bedtimes to achieve the prescribed TIB. We allowed flexibility in scheduling the TIB conditions because the 7 and 10 h TIB schedules could interfere with school assignments and extracurricular schedules. This flexibility prevented us from randomly assigning the order that each participant completed the schedules. Instead, order was included as a cofactor in statistical analyses. Actigraphy watch recordings and all night EEG recordings (see below) determined adherence to the prescribed schedules. If a participant’s TIB deviated by more than 1 hour from the prescribed TIB, the recording was rescheduled, or, if rescheduling was not possible, the data were excluded. Deviations causing data exclusion occurred in less than 1% of recordings.

### Daytime sleepiness and performance testing

Following four nights on the prescribed sleep schedule, participants reported to the lab for a full weekend day of performance and sleepiness testing. Participants arose at their habitual rise time and arrived at the lab at 0830. Any loose recording electrodes were reapplied, and subjects completed four test batteries at 2 hour intervals starting at 0900. All test batteries included a Karolinska Drowsiness Test/Alpha Attenuation Test (KDT/AAT), a psychomotor vigilance test (PVT), and a multiple sleep latency test (MSLT). The 1100 and 1500 batteries also included a Sternberg test of working memory. Participants also completed a Karolinska Sleepiness Scale (KSS) of subjective sleepiness and a positive and negative affect scale for children (PANAS-c). This report focuses on the KDT/AAT results and the relation of these results to executive functioning measured with the modified Sternberg test, waking vigilance measured with the PVT, and daytime sleepiness measured with the KSS and MSLT.

For each KDT/AAT, participants were seated comfortably at a desk facing a wall approximately one meter away with a dot on the wall at eye level. Participants initially focused on the dot for 3 min. They then closed their eyes for 2 min, focused on the dot for 2 min, and closed their eyes for another 2 min. Participants were asked to sit as still as comfortably possible, and EEG was recorded throughout the test. Participants marked the beginning and end of the test and each eyes open/closed change by pressing a push button that added an event marker to the EEG recording.

The KSS is a nine-point scale ranging from 1 “Very Alert” to 9 “Very Sleepy (fighting sleep). During each performance test battery, participants completed two KSS ratings, one prior to the start of the KDT/AAT and another 25 min later prior to the start of the MSLT.

During each test battery participants completed a 10 min PVT with intertrial intervals varying from 2 to 10 s. The log of the signal-to-noise ratio (LSNR) was the outcome measure of the PVT [12]. The more common measure, number of lapses greater than 500 ms is not appropriate for this dataset with children as young as 10 years of age who may have a mean reaction time greater than 500 ms. During each test battery participants completed an MSLT. Participants lay down in bed and made themselves comfortable. At lights off participants were instructed to try to fall asleep. The test concluded after 5 consecutive 20 second epochs of stage N1, or a single epoch of stage N2, N3, or REM sleep at which point the participant was awoken. If the participant did not fall asleep, the test was concluded after 20 min. Further PVT and MSLT details from this study have been previously published [3].

The 11 AM and 3 PM testing bouts included a modified Sternberg test of executive functioning. For each trial of the test a 2 or 4 member set of letters was displayed for participants to hold in memory. Then a probe letter was displayed, and participants were asked to respond as quickly and accurately as possible whether the probe letter was part of the memory set. The slope of the linear function describing the relation between reaction time and memory set size is a measure of working memory scanning efficiency [13]. Half of the negative probes, i.e., not in the memory set, were included in the preceding memory set. This recency modification to the Sternberg test adds an evaluation of the ability to resist proactive interference [14].

### EEG recording

On the second and fourth night of the prescribed TIB schedule, sleep EEG was recorded in the participants’ homes in their typical sleep environment. Sleep lab employees traveled to the homes and applied EEG electrodes at F3, F4, C3, C4, P3, P4, O1, and O2 with A1 and A2 mastoid electrodes. EOG electrodes were applied at LOC and ROC and referred to a central forehead electrode. Submental EMG was recorded bipolarly from the chin. Reference and ground electrodes were applied on the scalp and forehead. Upon waking after the fourth night, participants reported to the lab with electrodes still attached. Signals were recorded on Grass Aura24 ambulatory EEG recorders. All signals were recorded versus a reference electrode and signals such as C3-A2 were obtained by subtraction. Signals were digitized at 400 Hz. The Aura amplifiers have low frequency hardware filters with a −3 dB point at 0.5 Hz and a 6 dB/octave slope and high frequency hardware filters with a −3 dB point at 100 Hz and an 18 dB/octave slope.

### EEG analysis

Sleep stage scoring and spectral analysis details for the all night EEG recordings have been previously published [15]. For the analysis of waking EEG recorded during the KDT/AAT, analysis epochs were shortened to 5 s. Each epoch was analyzed with the FFT component of PASS Plus (Delta Software, St. Louis) using 2.56 s Welch tapered windows with a 1.31 second overlap yielding four windows per 5 s epoch. FFT resolution was 0.391 Hz. The analyses here focused on the alpha (8.01 Hz–11.91 Hz) EEG recorded from the occipital (O1-A2 and O2-A1) and central (C3-A2 and C4-A1) channels. Epochs containing artifacts were excluded from analysis after being marked with a computer program that identified low frequency movement artifacts and high frequency EMG artifacts. Power was averaged over all artifact free epochs for eyes open and separately for eyes closed for each of the 4 KDT/AAT sessions in a day. If fewer than six 5-s epochs were artifact free, data from that session were excluded (eyes open and eyes closed evaluated separately). The waking EEG power spectrum has a distinct peak in the alpha band. We determined which 0.391 Hz bin contained this peak. If the alpha power spectrum for an individual KDT trial did not show a distinct peak, the data for that trial were excluded from KDT peak analysis but were included in all other analyses.

The EEG data presented here will be shared in response to reasonable requests to the corresponding author.

### Statistical analyses

TIB and age effects on log alpha power were evaluated with mixed effects analysis. Mixed effects analysis is particularly suited for longitudinal studies because it accounts for the inherent correlations of multiple recordings from the same subject [16]. Analyses included TIB as a class measure with three levels (7, 8.5, 10 h) and age as continuous factor centered by subtracting the mean (13.3 y). The TIB effect differed significantly between subjects and was, therefore, also included as a random factor. Eyes open or closed was included as a class variable, and the analyses evaluated TIB, age, and eyes (open vs. closed) interactions. Rather than adding a fourth interaction term for electrode site, effects on O1, O2, C3, and C4 alpha EEG were tested in separate analyses. All analyses accounted for time of day effects. Analysis of the order effect that we have reported for the MSLT and PVT showed that this order effect was not significant for waking alpha measures; therefore, the order that participants completed the TIB conditions was not included as a cofactor. We also used mixed effects analysis to evaluate TIB, age, and eyes (open vs. closed) effects on the frequency at which alpha power peaks and the log of the magnitude of the peak.

In addition to evaluating the TIB effects on alpha EEG power, we tested the effects of total sleep duration recorded on the fourth night of the prescribed sleep schedule, as well as the effects of NREM and REM duration and N2 and N3 duration. We limited the sleep duration analyses to eyes closed alpha, and all analyses accounted for the effects of age and time of day.

The relation between subjective sleepiness ratings on the KSS and eyes closed alpha EEG power was evaluated with a mixed effects analysis that accounted for the effects of time of day. A separated analysis also accounted for the effects of night 4 sleep duration. A similar analysis evaluating the relation between PVT LSNR and eyes closed alpha EEG power also accounted for the order of the test because PVT performance decreased with repeated visits to the lab. Mixed effect analyses also tested the relation between eyes closed alpha EEG power and Sternberg test measured working memory scanning efficiency and proactive interference resistance. The relation between MSLT sleep likelihood and the eyes closed alpha power was evaluated with a nonlinear mixed survival analysis that determined the likelihood of falling asleep in each minute of the test and how alpha power affected this likelihood. The analysis accounted for effects of time of day, test order, and night 4 sleep duration on sleep likelihood.

Although the figures plot absolute alpha power, alpha power was log transformed for all statistical analyses.

## Results

Time in bed restriction effectively reduced sleep duration (*F*_2,149_ = 1559, *p* < .0001). Average (±standard error) night 4 sleep durations for the 7, 8.5, and 10 h TIB conditions were 406 ± 1, 472 ± 2, and 530 ± 2 min, respectively. The TIB effect on sleep duration did not change significantly with age (TIB × age interaction, *F*_2,464_ = 1.92, *p* = .15).

### TIB effects on waking alpha EEG

**Figure 1.**
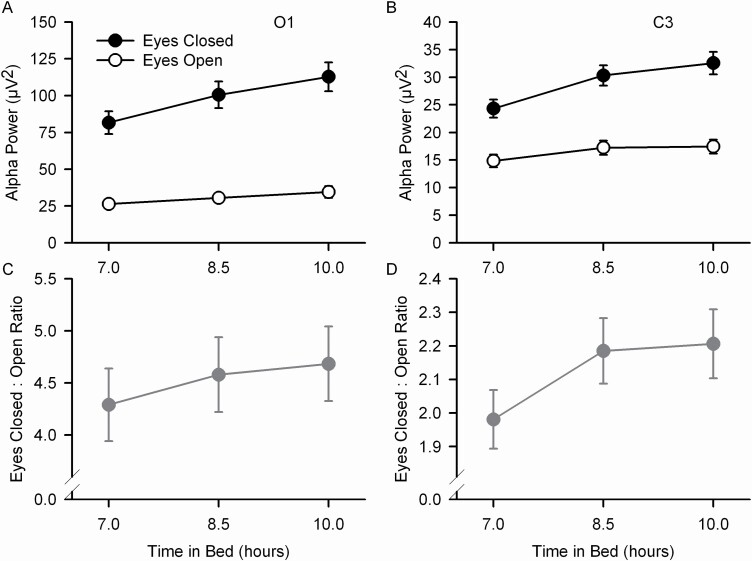
Mean (±s.e.) occipital (O1) and central (C3) EEG alpha power are plotted against time in bed for the eyes open (open circles) and eyes closed (filled circles) conditions and for the ratio of closed to open (filled gray circles and gray lines). For O1/A2 (A) and C3/A2 (B) EEG, decreasing time in bed reduced alpha power and decreased the difference between the eyes closed and eyes open conditions. The ratio of eyes closed to eyes open decreased as TIB was reduced for both O1 (C) and C3 (D).

Alpha power in both O1 and C3 decreased with TIB reduction (Figure 1A, Table 1). For both O1 and C3, alpha power was significantly smaller for 8.5 h than for 10 h TIB and smaller for 7 h than 8.5 h (Table 1). With eyes closed, alpha power was more than 200% greater than with eyes open for O1 (Figure 1A) and more than 60% greater for C3 (Figure 1B). The time in bed effect interacted with the eyes open/closed effect with the TIB effect being smaller for eyes open. However, analyzing the TIB effects separately for the eyes closed and eyes open recordings showed that alpha power decreased significantly (*p* < .0001 for all) for both eyes closed and open for both O1 and C3. The ratio of eyes closed to eyes open alpha power declined with TIB reduction (Figure 1C, O1, *F*_2,151_ = 7.74, *p* = .0006; Figure 1D, C3, *F*_2,151_ = 11.6, *p* < .0001).

**Table 1:** Mixed effects analysis results for time in bed (TIB), age, and eyes (open vs. closed) effects on occipital (O1) and central (C3) waking alpha power.

	O1		C3	
	*F*	*p*	*F*	*p*
TIB	*F*_2,152_ = 47.3	<.0001	*F*_2,151_ = 42.3	<.0001
TIB 7 vs. 10	*F*_1,152_ = 92.0	<.0001	*F*_1,151_ = 80.4	<.0001
TIB 7 vs. 8.5	*F*_1,152_ = 37.2	<.0001	*F*_1,151_ = 38.6	<.0001
TIB 8.5 vs. 10	*F*_1,152_ = 12.1	.0007	*F*_1,151_ = 7.51	.0069
Age	*F*_1,75_ = 30.9	<.0001	*F*_1,75_ = 15.0	.0002
Eyes	*F*_1,4286_ = 6562	<.0001	*F*_1,4295_ = 2639	<.0001
TIB × Age	*F*_2,141_ = 1.47	.23	*F*_2,141_ = 0.02	.98
TIB × Eyes	*F*_1,4286_ = 9.79	<.0002	*F*_1,4295_ = 9.44	<.0001
Age × Eyes	*F*_1,4286_ = 21.4	<.0001	*F*_1,4295_ = 0.16	.69
TIB × Age × Eyes	*F*_1,4286_ = 1.6	.20	*F*_1,4295_ = 0.59	.57

TIB effects on alpha power recorded from O2 and C4 were similar to those reported for O1 and C3 except that TIB effects on eyes open alpha power were not significant. See Supplementary Materials for details on O2 and C4 results.

### Age effects on waking alpha EEG

**Figure 2.**
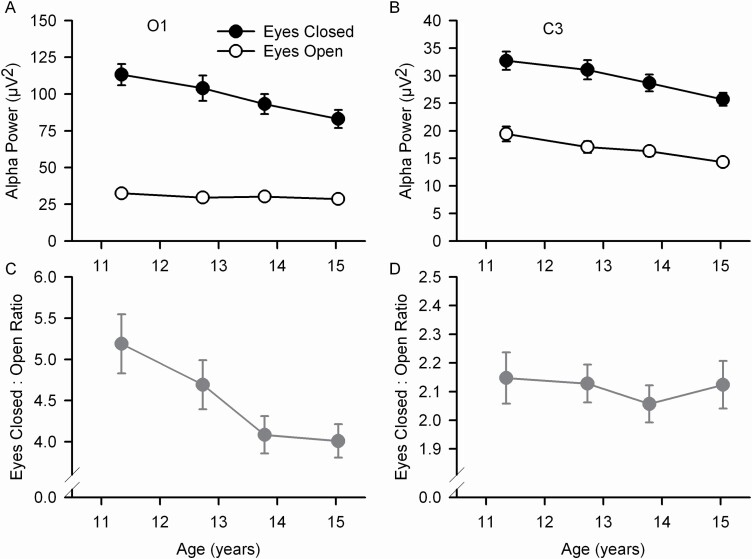
Mean (±s.e.) occipital (O1) and central (C3) EEG alpha power are plotted against quartile mean age for the eyes open (open circles) and eyes closed (filled circles) conditions and for the ratio of closed to open (filled gray circles and gray lines). For O1/A2 (A) and C3/A2 (B) EEG, alpha power declined across the 10–16 year age range. The ratio of eyes closed to eyes open decreased with age for O1 (C) but not for C3 (D).

For both O1 and C3, alpha power declined across the 10–16 year age range of this study (Figures 2A&B, Table 1). For O1 (*F*_1,4286_ = 21.4, *p* < .0001) but not for C3 (*F*_1,4295_ = 0.16, *p* = .69) the age and eyes effects interacted, with the age related decline in O1 alpha power being greater for eyes closed than for eyes open. The ratio of eyes closed to eyes open alpha power decreased with age for O1 (Figure 2C, *F*_1,75_ = 16.0, *p* < .0001) but not for C3 (Figure 2D, *F*_1,75_ = 2.71, *p* = .10). See Supplementary Materials for age effects on O2 and C4 alpha.

### TIB by age interaction

**Figure 3.**
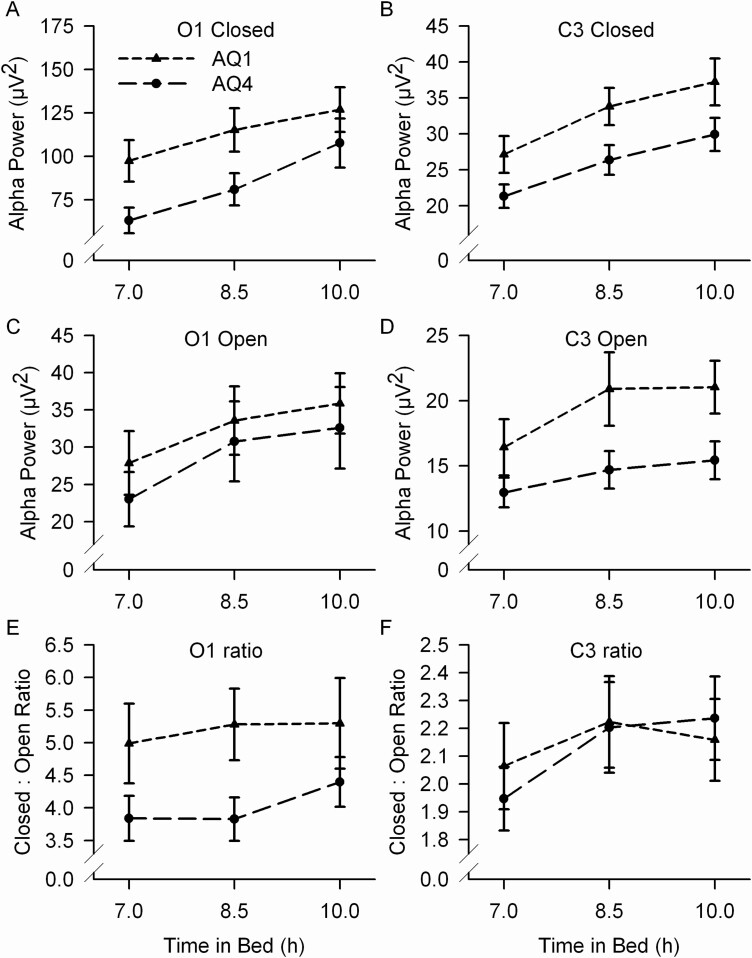
Mean (±s.e.) alpha power is plotted against TIB for the youngest age quartile (AQ1, mean age 11.3 y, short-dashed line, triangles) and oldest age quartile (AQ4, mean age 15.0 y, long-dashed line, circles) for eyes closed (A), eyes open (C) and the closed:open ratio (E) for O1 and for C3 (B, D, F). TIB and age effects did not interact significantly for either O1 or C3. TIB effects on the closed:open ratios did not change significantly with age.

Age and TIB effects on alpha power did not interact significantly for either O1 (Figures 3A and C, Table 1) or C3 (Figures 3B and D, Table 1). The TIB effect on the eyes closed/open ratio (Figures 3E and F) did not change significantly with age for either O1 (*F*_2,141_ = 1.31, *p* = .27) or C3 (*F*_2,141_ = 0.32, *p* = .73). There was no age-related change in the greater eyes closed effect seen with decreasing TIB, i.e. no three-way interaction eyes × TIB × age (O1, *F*_2,4286_ = 1.60, *p* = .20; C3, *F*_2,4295_ = 0.59, *p* = .57).

As shown in Supplementary Materials, for O2 EEG, the TIB and age effects interacted significantly (*F*_2,136_ = 3.81, *p* = .025) such that with increasing age there was an increase in the size of the power reduction as TIB was reduced from 8.5 to 7 h (*F*_1,136_ = 7.60 *p* = .0066).

### No sex differences in waking alpha EEG power

Adding a sex term to the analyses showed that alpha power did not differ between males and females (O1, *F*_1,4286_ = 0.44, *p* = .51; C3, *F*_1,4295_ = 0.05, *p* = .82). The reduction in alpha power with decreased TIB also did not differ by sex (O1, *F*_2,4286_ = 1.66, *p* = .19; C3, *F*_2,4295_ = 0.40, *p* = .67). The age-related alpha power decrease was steeper in males for O1 EEG (O1, *F*_1,4286_ = 5.14, *p* = .023) but not for C3 EEG (*F*_1,4295_ = 2.38, *p* = .12). No sex differences or sex interactions were found for O2 and C4 alpha (Supplementary Materials).

### Sleep stage duration effects on waking alpha EEG power

As described at the beginning of this Results section, sleep duration increased nearly linearly with increasing TIB. We evaluated the effects of night 4 sleep duration on eye closed alpha power, the measure that showed the greatest TIB effects, and the statistical analysis results are detailed in Table 2. O1 and C3 eyes closed alpha power both decreased significantly with decreasing total sleep duration, REM sleep duration, NREM sleep duration, and N2 sleep duration. O1 and C3 eyes closed alpha power were not related to night 4 N3 duration. The sleep duration effects increased with age for O1 but not for C3 (Table 2, TST by age interaction). The same analyses for O2, and C4 data produced similar results (Supplementary Materials).

**Table 2:** Mixed effects analysis results for sleep duration effects on eyes closed occipital (O1) and central (C3) waking alpha power.

	O1		C3	
	*F*	*p*	*F*	*p*
Total Sleep Time	*F*_1,76_ = 61.7	<.0001	*F*_1,76_ = 101	<.0001
REM Sleep Time	*F*_1,76_ = 29.0	<.0001	*F*_1,76_ = 48.9	<.0001
NREM Sleep Time	*F*_1,76_ = 34.3	<.0001	*F*_1,76_ = 86.9	<.0001
N2 Sleep Time	*F*_1,76_ = 68.8	<.0001	*F*_1,76_ = 93.1	<.0001
N3 Sleep Time	*F*_1,76_ = 1.77	.19	*F*_1,76_ = 1.40	.24
TST X Age	*F*_1,1906_ = 5.79	.016	*F*_1,1909_ = 0.47	.49

### TIB and age effects on the waking alpha EEG power spectrum

**Figure 4.**
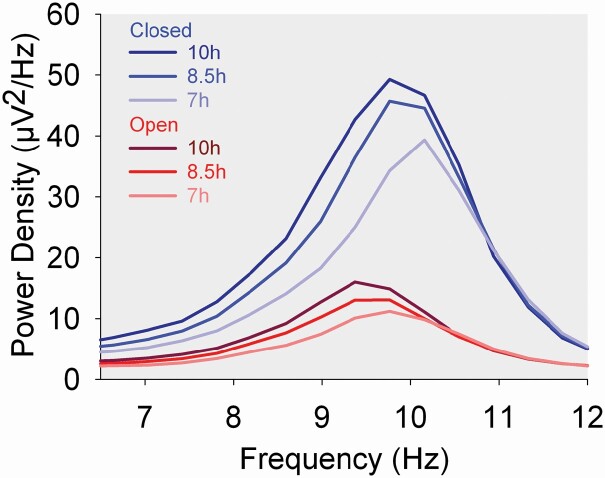
O1/A2 EEG alpha power spectra for the three time in bed conditions for the eyes closed (darkening shades of blue) and eyes open (darkening shades of red) conditions. For each 0.39 Hz frequency bin, mean power density is plotted against the midpoint of the bin. The frequency at which alpha power reached a peak was higher for the eyes closed than for the eyes open condition. For both the eyes closed and eyes open conditions, the alpha peak frequency increased as TIB was reduced.

**Figure 5.**
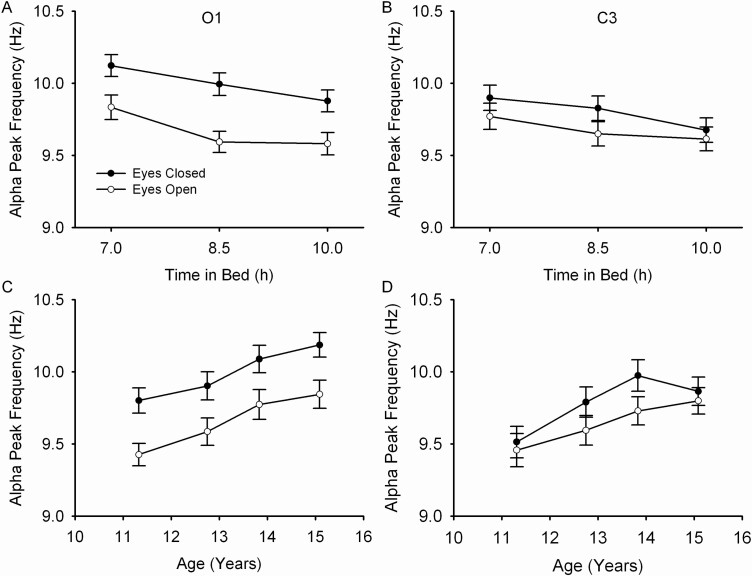
Mean (±s.e.) alpha peak frequency is plotted against TIB for O1 (A) and C3 (B). Mean (±s.e.) alpha peak frequency is plotted against quartile mean age for O1 (C) and C3 (D). For both the eyes closed (filled circles) and eyes open (open circles) conditions, alpha peak frequency increased as TIB was reduced and increased with increasing age.

The amplitude of the power peak within the alpha band (Figure 4) was greater with eyes closed (O1, *F*_1,3932_ = 5185, *p* < .0001; C3, *F*_1,3416_ = 1411, *p* < .0001), and decreased with decreasing TIB (O1, *F*_2,148_ = 48.6, *p* < .0001; C3, *F*_1,146_ = 28.9, *p* < .0001). The amplitude of the peak also declined with age for O1 (*F*_1,74 =_ 14.2, *p* = .0002) but not for C3 (*F*_1,74_ = 3.65, *p* = .060). For O1, the frequency at which the alpha peak occurred was 0.31 ± 0.02 Hz (Mixed effect estimate ± standard error) greater with eyes closed than with eyes open (*F*_1,3932_ = 424, *p* < .0001). As shown in Figures 4 and 5A, O1 alpha peak frequency increased as TIB decreased (*F*_2,148_ = 37.6, *p* < .0001). The O1 alpha peak frequency increased by 0.039 ± 0.018 Hz for each additional year of age (Figure 5C, *F*_1,74_ = 4.68, *p* = .038). The TIB effect did not change with age (*F*_2,139_ = 0.68, *p* = .51). Results were similar for C3: the frequency of the alpha peak was higher with eyes closed (*F*_1,3416_ = 74.7, *p* < .0001). It increased with TIB restriction (Figure 5B, *F*_2,145_ = 44.4, *p* < .0001), but did not change significantly with age (Figure 5D, *F*_1,74_ = 2.22, *p* = .14). For C3 as well, TIB and age effects on the peak frequency did not interact (*F*_2,131_ = 1.47, *p* = .23). See Supplementary Materials for effects on the O2 and C4 power spectra.

### Relation of alpha EEG to daytime sleepiness, vigilance, and executive function

**Figure 6.**
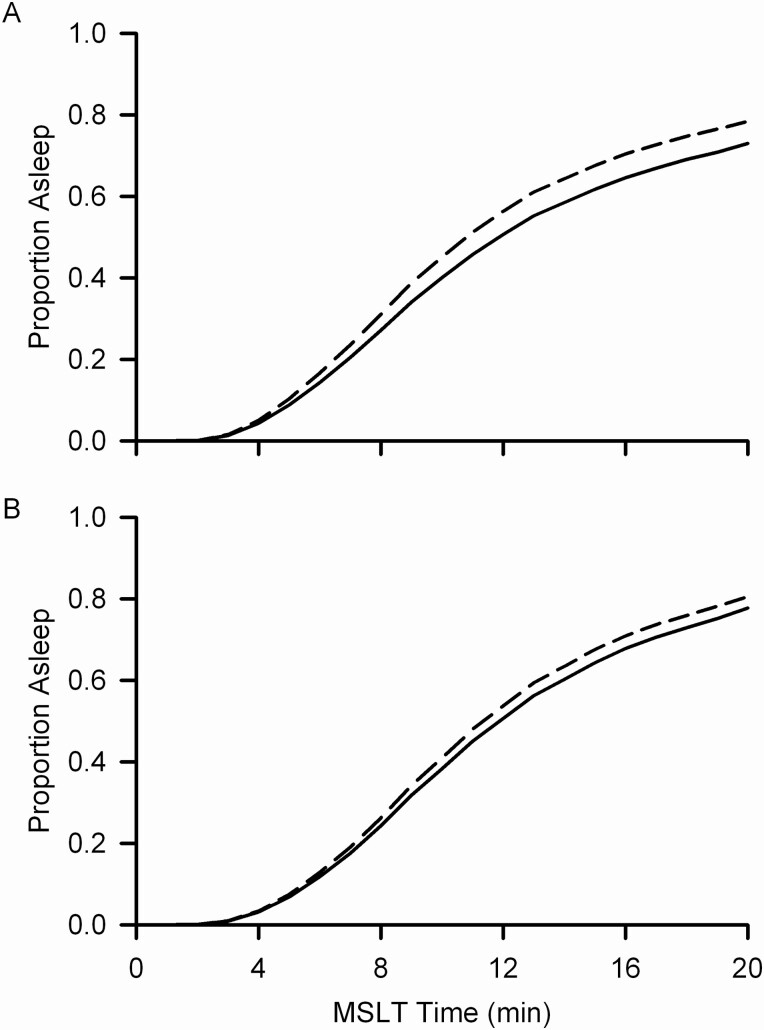
Effect of log alpha power on multiple sleep latency test (MSLT) sleep likelihood. The predicted (result of mixed effects survival analysis) proportion of subjects asleep is plotted against minute of the MSLT. (A) The solid line shows sleep likelihood adjusted for the effect of log alpha power equal to the mean for 10 h TIB, and the dashed line shows it adjusted for the effect of log alpha power equal to the mean for 7 h TIB. The likelihood of falling asleep decreased slightly but significantly (*p* < .0001) with increasing log alpha power. (B) Similar to A but the analysis accounted for the effect of night 4 sleep duration on MSLT sleep likelihood.

**Figure 7.**
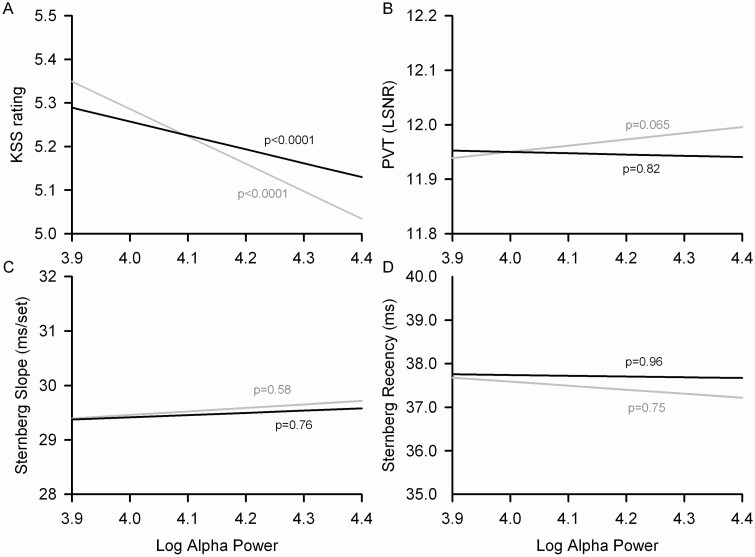
Mixed effects analysis estimates of the linear relations between daytime sleepiness/ performance and log alpha power are plotted for KSS ratings (A), PVT log signal to noise ratio (B), slope of the relation between Sternberg reaction time and memory set size (C), and Sternberg reaction time difference between recent and non-recent probes (D). Each plot includes a line for the relation to log alpha power with age and time of day effects accounted for (gray) and a separate line for the relation to log alpha power with total sleep time (TST), age, and time of day effects accounted for (black). PVT and Sternberg analyses also account for an order effect. The range of the *x*-axis extends slightly beyond the mean log alpha power for 7 h in bed (3.91) and for 10 h in bed (4.32).

Objective sleepiness measured as the likelihood of falling asleep during the MSLT decreased significantly (*t*_75_ = −7.56, *p* < .0001) with increasing O1 eyes closed alpha power (Figure 6A) even when accounting for the significant (*t*_75_ = −19.0, *p* < .0001) effect of the prior night’s sleep duration (Figure 6B). Subjective sleepiness ratings on the KSS decreased significantly (*F*_1,2254_ = 132, *p* < .0001) with increasing O1 eyes closed alpha power (Figure 7A). This relation between subjective sleepiness and alpha power persisted (*F*_1,1957_ = 22.6, *p* < .0001) even when accounting for the significant (*F*_1,1957_ = 607, *p* < .0001) effect of the prior night’s sleep duration. These significant effects of alpha power on daytime sleepiness were small. The reduction in average log alpha power as time in bed was reduced from 10 to 7 h was associated only with a 6 percentage point increase in the likelihood of falling asleep by the 20th minute of the MSLT (Figure 6A) and only a ¼ point increase in KSS rating (Figure 7A).

With effects of time of day, age, and order accounted for, sustained vigilance measured as the log of the signal to noise ratio on the PVT showed a trend (*F*_1,2233_ = 3.41, *p* = .065) toward increasing with increasing O1 eyes closed alpha, but this relation was not significant (*F*_1,1965_ = 0.05, *p* = .82) once the effect of prior night’s sleep duration was accounted for (Figure 7B). Working memory scanning efficiency (Figure 7C) measured with the Sternberg test was not significantly (*F*_1,1066_ = 0.30, *p* = .58) associated with O1 eyes closed alpha power, nor was the ability to resist proactive interference (Figure 7D, *F*_1,1066_ = 0.10, *p* = 0.75). Relations between C3, O2, and C4 alpha power and daytime sleepiness, vigilance, and executive functioning are presented in the Supplementary Material.

Post hoc analyses of neighboring frequency bands, theta (4–8 Hz), and low beta (12–23 Hz), determined whether the relations between waking EEG activity and daytime sleepiness were unique to the alpha frequency band. With effects of sleep duration and time of day accounted for, KSS ratings were related to O1 eyes closed low beta (12–23 Hz, *F*_1,1958_ = 16.4, *p* < .0001) power but not O1 eyes closed theta (4–8 Hz, *F*_1,1958_ = 1.75, *p* = .19) power. With effects of sleep duration, order, and time of day accounted for, the likelihood of falling asleep during the MSLT decreased with increasing O1 eyes closed theta power (*t*_75_ = −3.89, *p* = .0002) and with increasing O1 eyes closed low beta power (*t*_75_ = −5.92, *p* < .0001).

## Discussion

These results expand on our prior observation [7] that insufficient sleep alters waking brain physiology. Focusing on alpha, the frequency band our prior study found to be most strongly affected by sleep loss, we evaluated the effects of sleep loss and age as well as their interaction. We examined effects of sleep loss and age on the power spectrum within the alpha band. We also used the sleep restriction related changes in alpha power to determine whether daytime sleepiness, vigilance, and executive function are related alpha EEG activity. Our findings bear on two important neuroscience issues: how does sleep need change over adolescence, and how does insufficient sleep affect waking brain function.

### Alpha power decrease with TIB restriction

Alpha power with eyes closed decreased with TIB restriction as did the ratio of eyes closed to eyes open alpha power (alpha attenuation coefficient—AAC). These findings are similar to what was previously found with sleep loss in adults [4–6]. We previously reported [7] for the current adolescent dataset that TIB restriction decreases waking EEG power not only in the 8–12 Hz band but also in 1–4, 4–8, and 12–17 Hz. We interpreted this wide-spread decrease as an indicator of insufficient sleep recuperation which impairs waking brain activity. As we noted, the insufficient recovery cannot be attributed to decreasing the recovery processes of slow wave sleep because our imposed TIB reduction reduced the terminal hours of sleep which contain little slow wave EEG. Indeed, our current analyses of the effects of night 4 sleep stage durations showed that alpha power was not related to the small amounts of stage N3 in the terminal hours of sleep but was significantly related to prior N2 and REM duration. To clarify, we are not implying that N3 sleep plays no role in the recovery of waking brain activity. Indeed, TIB restriction severe enough to decrease N3 duration would certainly have an even greater detrimental impact. Instead, our data show that TIB restriction that decreases only N2 and REM duration is sufficient to alter waking EEG activity. This finding contradicts our own previous view [17] and those of the two process model [18] that attribute recuperative or homeostatic properties to slow wave sleep alone. TIB restriction by curtailing the terminal few hours in bed will necessarily decrease both N2 and REM sleep, and it would be difficult to disentangle the contribution of N2 and REM to recovery of optimal waking brain activity. Recent sleep research (including contributions from our lab) has focused on recovery during NREM sleep, but REM may also play an important role. Indeed, it would be astonishing if the huge amounts of a normal sleep period regularly devoted to REM throughout adult life (~20%) did not play an important biological role in sleep recovery functions.

In adults, sleep deprivation caused an increase in alpha power with eyes open [4]. In our adolescent recordings sleep restriction decreased alpha power in both the eyes closed (significant for all channels) and eyes open (significant for O1 and C3 but not O2 and C4) conditions. The different response of adolescents in our study may be related to the magnitude of sleep loss (total sleep deprivation in adults versus a 3 hour decrease in TIB in the current study). Indeed, Akerstedt et al. found that eyes open alpha power was only increased at high levels of subjective sleepiness, 7 or greater on the Karolinska sleepiness scale [4]. A more remote possibility is that the difference between adolescents and adults indicates a physiological susceptibility of the less mature brain of adolescents.

### Alpha power decreases with age

The age-related decrease in waking EEG alpha power appears to be part of a general decrease in EEG power during adolescence across a wide range of frequency bands. This decrease is more marked in the slower frequencies [19]. Fine band analysis has found an increase in power in the higher alpha frequencies 10–12 Hz [20], but this increase resulted from a shift in the peak alpha frequency (see below). We have previously interpreted the decline in sleep EEG power across adolescence in delta (1–4 Hz), theta (4–8 Hz), and sigma (11–15 Hz) as being indicators of adolescent brain maturation driven by synaptic pruning [21, 22]. Synaptic density peaks in childhood and decreases steeply across adolescence [23] as does cortical metabolic rate [24]. The EEG at the scalp is the sum of simultaneous membrane potential oscillations of cortical pyramidal cells. Synaptic pruning would decrease the size of the pool of neurons oscillating in unison and thereby decrease EEG amplitude. However, Miskovic et al. [25] note that state-dependent differences in the maturational trends for EEG power argue against structural alterations as the only cause of the age-related decline in EEG power. Slow wave EEG declines linearly with age for wake and REM sleep, but for NREM sleep slow wave EEG power is stable across late childhood before declining steeply across adolescence [22]. It remains possible that the adolescent decline in EEG power results from cortical network reorganization in addition to synapse elimination.

### Interaction of TIB and age effects on alpha power

We had hypothesized that the brain maturation of adolescence would decrease sleep need, resulting in smaller sleep restriction effects on waking alpha power. The data here do not support that hypothesis. Thus, the effect of TIB on the alpha attenuation coefficient, i.e. the eyes closed to eyes open alpha power ratio, did not change with age, nor did the TIB effect on O1, C3, or C4 alpha power. The only significant interaction of age and TIB effects was the opposite of our prediction. For O2, the reduction in alpha power for 7 hour TIB versus 8.5 hour TIB increased with age. Furthermore, for both occipital channels (but not the central channels), there was an *increase* in the slope of the relation between alpha power and prior sleep duration. Although the mixed results may not be sufficient to conclude that sleep need increases, the effects of sleep restriction on waking alpha power certainly do not indicate a declining sleep need across adolescence. This finding differs from MSLT results of the same dataset, where TIB restriction produced a greater increase in daytime sleepiness in younger subjects [3, 11]. However, the current findings agree with daytime vigilance data from this dataset, where impairment of PVT performance with TIB restriction did not change significantly with age [3]. Thus far, the divergence of these results complicates our goal of providing recommended age-specific sleep durations for adolescents. We are currently recording data on 16–22-year-old participants and will be able to determine if these different measures of sleep need converge at the end of adolescence.

### Age and TIB effects on peak alpha frequency

The increase in alpha frequency across childhood and into adolescence was documented in early EEG studies [26, 27]. Our findings agree with recent findings of an adolescent increase in peak alpha frequency [20]. Increased white matter via axonal myelination has been proposed as the mechanism producing the frequency increase. Miskovic et al.’s [25] finding of an age-related increase in the strength of long-range connectivity supports this interpretation. The increase in alpha peak frequency with decreasing TIB is contrary to our expectations. In adults, sleep deprivation produces a slowing of alpha toward theta frequencies and an increase in slow wave (delta) EEG [4]. We predicted that sleep restriction in adolescents would also produce an EEG slowing evident in a decrease in the alpha peak frequency. Within individuals, alpha peak frequency increases when engaged in a task [28]. Sleep loss might increase peak frequency by preventing the brain from fully disengaging from prior activity. Prior studies have not reported whether sleep deprivation affected peak alpha frequency; therefore, further data are needed on this sleep loss response in both adolescents and adults.

### Alpha EEG relation to daytime sleepiness, vigilance, and executive function

Alpha rhythms are thought to play a role in cognitive processes such as attention and working memory [8–10]. We did not find a relation between alpha and cognitive processes. Waking EEG alpha power was not related to sustained attention measured on a PVT shortly after the EEG recording or working memory scanning efficiency measured with a Sternberg test shortly before the EEG recording. A current theory for the function of alpha waves proposes that alpha waves are involved in the inhibition of information irrelevant to the task at hand [29]. However, we found no relation between alpha activity and the modified Sternberg test recency measure of the ability to resist proactive interference.

Waking alpha EEG power was related to both objective and subjective measures of daytime sleepiness even with the effects of prior sleep duration accounted for. This finding confirms prior observations that alpha activity can be used as an indicator of subjective daytime sleepiness [6] and is consistent with the decrease in alpha activity across a period of sleep deprivation [5]. Our post hoc analyses demonstrated that this relation between waking EEG power and daytime sleepiness is not limited to the alpha frequency band and may, instead, be a result of suppression of brain activity across a wide range of EEG frequencies.

### Future directions

The findings here suggest at least four broad areas for further research. As we noted in our prior report, it would be interesting to determine whether the depression of waking EEG power by reduced sleep durations is caused by decreased wave amplitude or wave incidence in the affected frequency bands. This basic distinction is opaque to Fourier analysis but can be made with other methods, such as period-amplitude analysis [30]. A second broad research area, also one we previously mentioned, is whether the depression of waking EEG power could be developed into a test for sleep sufficiency for first responders and other personnel for whom sustained vigilance is critical. A third research area pointed to by our findings would be a more detailed analysis of the underlying neuro-metabolic mechanism using the depression of EEG power as an investigatory tool. We believe that pursuits of sleep and EEG research on these diverse levels of integration could importantly advance our knowledge of the function of human sleep. Finally, to address some of the questions left unanswered by the current study, it is critical to extend beyond the 10–16 year old age range.

### Limitations

As we have noted in prior publications, subject recruitment was biased against participants who need long habitual sleep duration. During recruitment several participants, or the participants’ parents, declined to enroll in the study when they learned that one condition required maintaining a 7-hour time in bed schedule for four consecutive nights. Several enrolled participants actually withdrew during the first recording due to negative impacts of reduced sleep duration. The TIB effects on waking EEG might have been even stronger if participants needing a long sleep duration were included in the subject pool. Alpha EEG activity is greatest with the mind unfocused and relaxed. Our instructions for the EEG recordings did not include any specific request to clear the mind. Different levels of focused thought or attention may have therefore affected the TIB effects reported here. Most of the results presented here were similar for occipital and central EEG and for left and right hemispheres; however, there were some differences such as the significant TIB effect on eyes open alpha power for O1 and C3 but not O2 and C4 as well as the significant TIB by age interaction for O2 but no other signal. Additional data are needed to determine if these differences are replicable and meaningful.

## Conclusions

Sleep loss affects waking alpha EEG activity by decreasing power and increasing the frequency at which alpha power is at its maximum. We interpret the alpha power decrease as an EEG indicator of prior insufficient sleep dependent recuperation. As we previously noted, this finding points to the potential of using waking EEG power as an easily obtained objective indicator of prior sleep sufficiency. A priori, we proposed that the age-related changes in the relation between sleep duration and waking alpha power could be used as a measure of whether sleep need changes across adolescence. This measure does not indicate a decrease in sleep need across ages 10–16 years.

## Supplementary Material

zpac015_suppl_Supplementary_Materials

## Notes

Work performed at University of California Davis, USA.
