# Supplementary material for: Sleep restriction and age effects on waking alpha EEG activity in adolescents
Source: Sleep Adv. 2022 May 10;3(1):zpac015. doi: 10.1093/sleepadvances/zpac015 (PMC9154075; doi:10.1093/sleepadvances/zpac015)
Supplement: zpac015_suppl_Supplementary_Materials [file zpac015_suppl_supplementary_materials.zip › zpac015_suppl_Supplementary_Results.docx]

**Supplemental Results**

TIB effects on waking alpha EEG (O2 and C4)

Eyes closed alpha power significantly exceeded eyes open alpha power for both O2 and C4 (Fig. 1S; O2. F_1,4138_=6571, p<0.0001; C4. F_1,4196_=2953, p<0.0001). Alpha power decreased with TIB reduction (Figure 1S_A, O2 F_2,151_=4.65 p=0.011; Figure 1S_B, C4 F_2,151_=3.89, p=0.022) with only the 7 vs. 10 h TIB contrast significant for O2 (F_1,151_=9.17, p=0.0029) and both the 7 vs. 8.5 (F_1,151_=4.48, p=0.036) and 7 vs. 10 h TIB (F_1,151_=6.92, p=0.0094) contrasts significant for C4. The time in bed effect interacted with the eyes open/closed effect (O2 F_2,4138_=9.49, p<0.0001; C4 F_2,4196_=10.8, p<0.0001) with the TIB effect being smaller for eyes open. Analyzing the TIB effects separately for the eyes closed and eyes open recordings showed that alpha power decreased significantly for eyes closed (p<0.0005 for both O2 and C4) but not for eyes open (p>0.15 for both O2 and C4). The ratio of eyes closed to eyes open alpha power declined with TIB reduction (Fig. 1S_C, O2, F_2,151_=7.37, p=0.0009; Fig. 1S_D, C4, F_2,151_=11.9, p<0.0001).

Age effects on waking alpha EEG (O2 and C4)

For both O2 (F_1,75_=28.9, p<0.0001) and C4 (F_1,75_=16.1, p=0.0001), alpha power declined across the 10 to 16 year age range of this study (Figs. 2S_A&B). For O2 (F_1,4138_=11.1, p=0.0009) but not for C4 (F_1,4196_=2.57, p=0.11) the age and eyes effects interacted, with the age related decline in O2 alpha power being greater for eyes closed than for eyes open. The ratio of eyes closed to eyes open alpha power decreased with age for O2 (Fig 2C, F_1,75_=12.3, p=0.0008) but not for C4 (Fig. 2D, F_1,75_=1.61, p=0.21).

TIB by age interaction (O2 and C4)

Age and TIB effects on alpha power interacted significantly (F_2,136_=3.81, p=0.025) for O2 alpha (Fig. 3S_A&C) with the 7 vs. 8.5 hour effect increasing with age (F_1,136_=7.60, p=0.0066). Age and TIB effects did not interact significantly (F_2,137_=0.98, p=0.38) for C4 (Figs. 3S_B & D). The TIB effect on the eyes closed/open ratio (Figs. 3E & F) did not change significantly with age for either O2 (F_2,135_=0.1, p=0.90) or C4 (F_2,137_=0.05, p=0.95). There was no age-related change in the greater eyes closed effect seen with decreasing TIB, i.e. no 3 way interaction eyes X TIB X age (O2, F_2,4138_=0.09, p=0.92; C4, F_2,4196_=0.18, p=0.83).

No Sex Differences in waking alpha EEG power (O2 and C4)

Adding a sex term to the analyses showed that alpha power did not differ between males and females (O2, F_1,4138_=0.81, p=0.37; C4, F_1,4196_=0.33, p=0.57). The reduction in alpha power with decreased TIB also did not differ by sex (O2, F_2,4138_=0.35, p=0.71; C4, F_2,4196_=0.08, p=0.92). The age-related alpha power decrease did not differ between sexes for either O2 (F_1,4138_=2.09, p=0.15) or C4 (F_1,4196_=1.79, p=0.18).

Sleep Stage Duration Effects on waking alpha EEG power (O2, C3, and C4)

O2, C3, and C4 eyes closed alpha power decreased significantly (O2, F_1,76_=55.4, p<0.0001; C3, F_1,76_=101, p<0.0001; C4, F_1,76_=57.2, p<0.0001) with decreasing sleep duration. Effects were also significant for durations of REM sleep (O2, F_1,76_=25.5, p<0.0001; C3, F_1,76_=48.9, p<0.0001; C4, F_1,76_=27.1, p<0.0001), NREM sleep (O2, F_1,76_=48.2, p<0.0001; C3, F_1,76_=86.9, p<0.0001; C4, F_1,76_=51.6, p<0.0001), and stage N2 (O2, F_1,76_=51.2, p<0.0001; C3, F_1,76_=93.1, p<0.0001; C4, F_1,76_=66.7, p<0.0001), but not for stage N3 duration (O2, F_1,76_=1.69, p=0.20; C3, F_1,76_=1.40, p=0.24; C4, F_1,76_=1.06, p=0.31).

TIB and Age effects on the waking alpha EEG power spectrum

The amplitude of the power peak within the alpha band (Fig. 4S) was greater with eyes closed (O2, F_1,3770_=5122, p<0.0001; C4, F_1,3387_=1420, p<0.0001), and decreased with decreasing TIB (O2, F_2,147_=33.4, p<0.0001; C4, F_1,145_=19.4, p<0.0001). The amplitude of the peak also declined with age for O2 (F_1,74_=11.6, p=0.0011) and C4 (F_1,73_=5.06, p=0.028). For O2, the frequency at which the alpha peak occurred increased by 0.36 +/- 0.03 Hz (Mixed effect estimate +/- standard error) with eyes closed compared to eyes open (F_1,3770_=550, p<0.0001). As shown in Figs. 4S and 5S_A, O2 alpha peak frequency increased as TIB decreased (F_2,147_=51.7, p<0.0001). The O2 alpha peak frequency increased by 0.026 +/- 0.022 Hz for each additional year of age (Fig 5S_C, F_1,74_=4.94, p=0.029). The TIB effect did not change with age (F_2,132_=2.34, p=0.10). Results were similar for C4: the frequency of the alpha peak was higher with eyes closed (F_1,3387_=41.8, p<0.0001). It increased with TIB restriction (Fig. 5S_B, F_2,145_=27.3, p<0.0001), but did not change significantly with age (Fig 5S_D, F_1,73_=0.62, p=0.43). For C4 as well, TIB and age effects on the peak frequency did not interact (F_2,125_=0.03, p=0.97).

Relation of alpha EEG to daytime sleepiness, vigilance, and executive functioning

Even when accounting for the significant effect of the prior night’s sleep duration, subjective sleepiness ratings on the KSS decreased significantly with increasing O2, C3, and C4 eyes closed alpha power (O2, F_1,1910_=10.4, p=0.0013; C3, F_1,1960_=18.4, p<0.0001; C4, F_1,1930_=12.3, p=0.0005). Objective sleepiness measured as the likelihood of falling asleep during the MSLT also decreased significantly with increasing O2, C3, and C4 eyes closed alpha power (O2, t_75_=-8.22, p<0.0001; C3, t_75_=-4.38, p<0.0001; C4, t_75_=-4.76, p<0.0001) even when accounting for the significant effect of the prior night’s sleep duration. With time of day, age, and order effects accounted for, sustained vigilance measured as the log of the signal to noise ratio on the PVT was not related to O2, or C4 eyes closed alpha power (O2, F_1,2162_=0.00, p=0.95; C4, F_1,2191_=1.28, p=0.26). PVT performance was significantly (F_1,2240_=5.21, p=0.023) related to C3 alpha power, but this relation was not significant (F_1,1968_=0.36, p=0.55) once the effect of night 4 sleep duration was accounted for. Working memory scanning efficiency measured with the Sternberg test was not significantly associated with eyes closed alpha power (O2, F_1,1025_=2.24, p=0.13; C3, F_1,1075_=0.30, p=0.59; C4, F_1,1046_=0.01, p=0.91), nor was the ability to resist proactive interference (O2, F_1,1025_=2.46, p=0.1255; C3, F_1,1075_=0.38, p=0.54). The ability to resist proactive interference improved significantly with increasing C4 alpha power (F_1,1046_=4.67, p=0.031) and this result persisted when accounting for sleep duration effects (F_1,908_=4.26, p=0.039). As a single positive result at alpha=0.05 among many tests, this positive relation between C4 alpha power and executive functioning would have to be replicated before concluding that it is meaningful.

**Supplement Figure Captions**

Figure 1S. Mean (+/- s.e.) occipital (O2) and central (C4) EEG alpha power are plotted against time in bed for the eyes open (open circles) and eyes closed (filled circles) conditions and for the ratio of closed to open (filled gray circles and gray lines). For O2/A1 (A) and C4/A1 (B) EEG, decreasing time in bed reduced alpha power and decreased the difference between the eyes closed and eyes open conditions. The ratio of eyes closed to eyes open decreased as TIB was reduced for both O2 (C) and C4 (D).

Figure 2S. Mean (+/- s.e.) occipital (O2) and central (C4) EEG alpha power are plotted against quartile mean age for the eyes open (open circles) and eyes closed (filled circles) conditions and for the ratio of closed to open (filled gray circles and gray lines). For O2/A1 (A) and C4/A1 (B) EEG, alpha power declined across the 10 to 16 year age range. The ratio of eyes closed to eyes open decreased with age for O2 (C) but not for C4 (D).

Figure 3S. Mean (+/- s.e.) alpha power is plotted against TIB for the youngest age quartile (AQ1, mean age 11.3 y, short dashed line, triangles) and oldest age quartile (AQ4, mean age 15.0 y, long dashed line, circles) for eyes closed (A), eyes open (C) and the closed:open ratio (E) for O2 and for C4 (B, D, F). TIB and age effects did not interact significantly for C4 but did for O2 with the 7 vs. 8.5 h difference increasing with age. TIB effects on the closed:open ratios did not change significantly with age.

Figure 4S. O2/A1 EEG alpha power spectra for the three time in bed conditions for the eyes closed (darkening shades of blue) and eyes open (darkening shades of red) conditions. For each 0.39 Hz frequency bin, mean power density is plotted against the midpoint of the bin. The frequency at which alpha power reached a peak was higher for the eyes closed than for the eyes open condition. For both the eyes closed and eyes open conditions, the alpha peak frequency increased as TIB was reduced.

Figure 5S. Mean (+/- s.e.) alpha peak frequency is plotted against TIB for O2 (A) and C4 (B). Mean (+/- s.e.) alpha peak frequency is plotted against quartile mean age for O2 (C) and C4 (D). For both the eyes closed (filled circles) and eyes open (open circles) conditions, alpha peak frequency increased as TIB was reduced and increased with increasing age.
