# Supplementary figures and images for: Sleep restriction and age effects on waking alpha EEG activity in adolescents
Source: Sleep Adv. 2022 May 10;3(1):zpac015. doi: 10.1093/sleepadvances/zpac015 (PMC9154075; doi:10.1093/sleepadvances/zpac015)

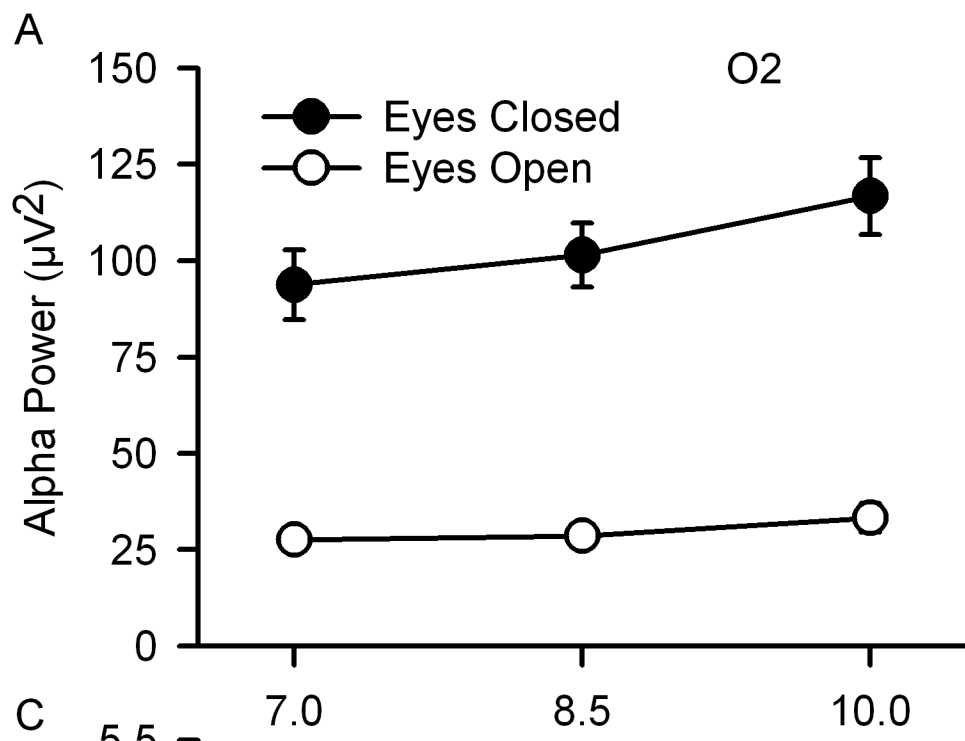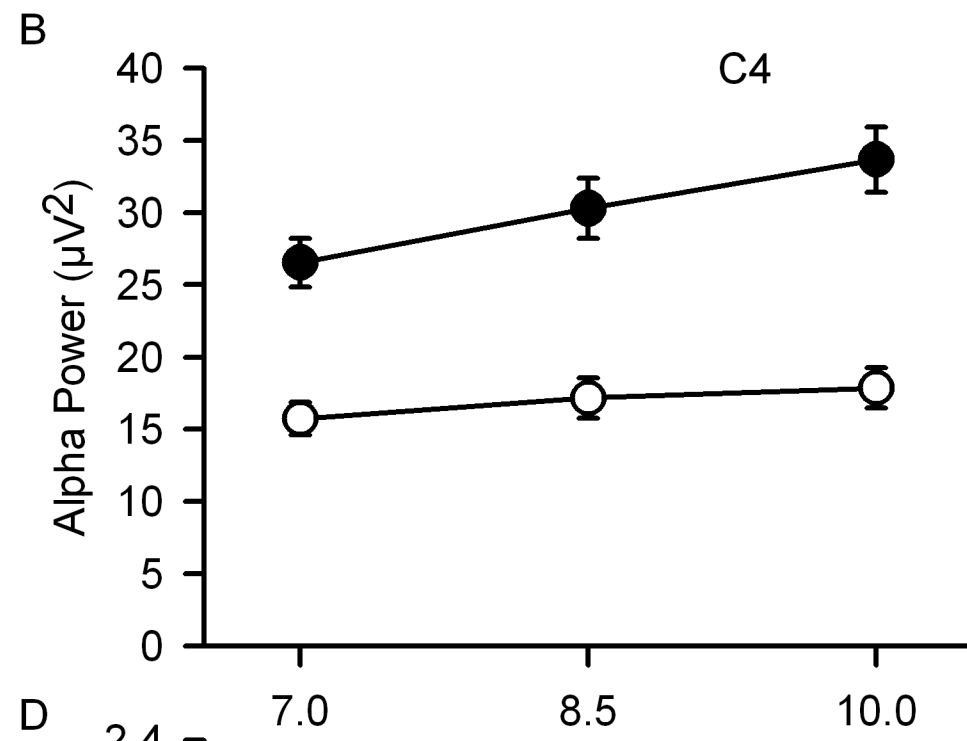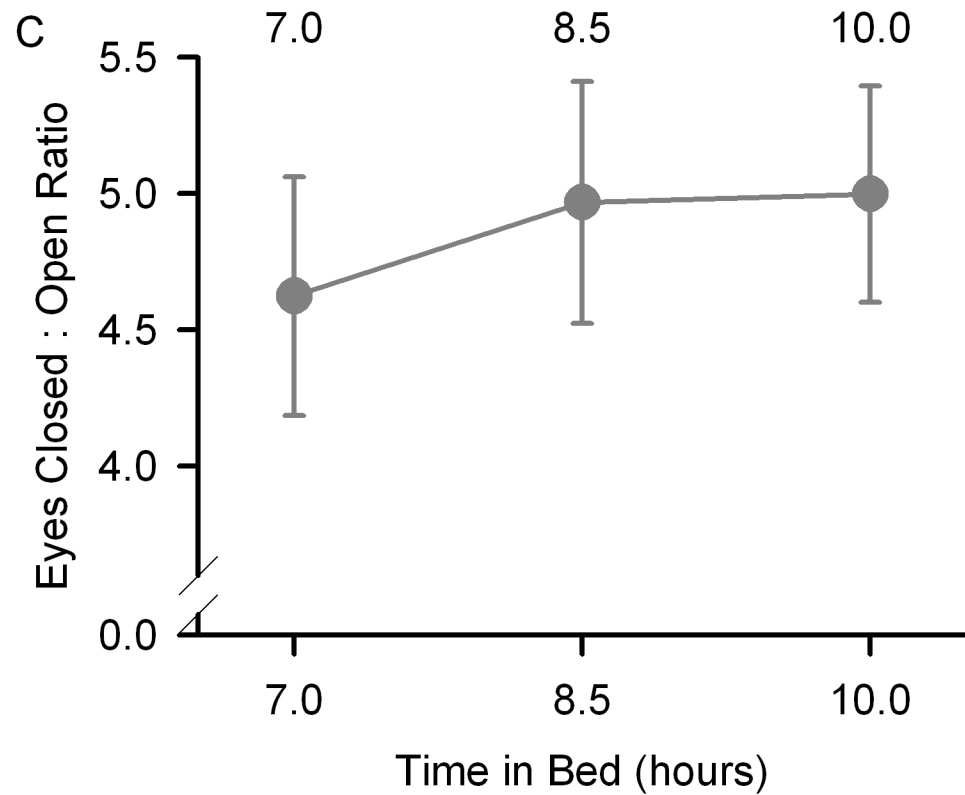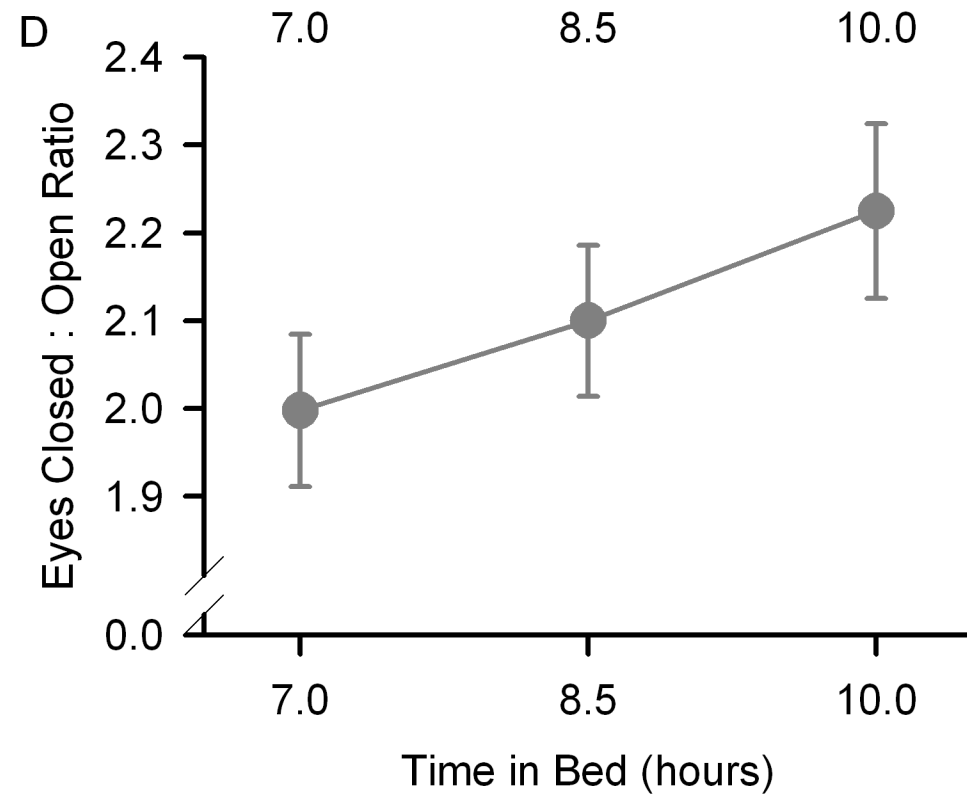

Supplement: zpac015_suppl_Supplementary_Materials [file zpac015_suppl_supplementary_materials.zip › zpac015_suppl_Supplementary_Figure_S1.PDF]

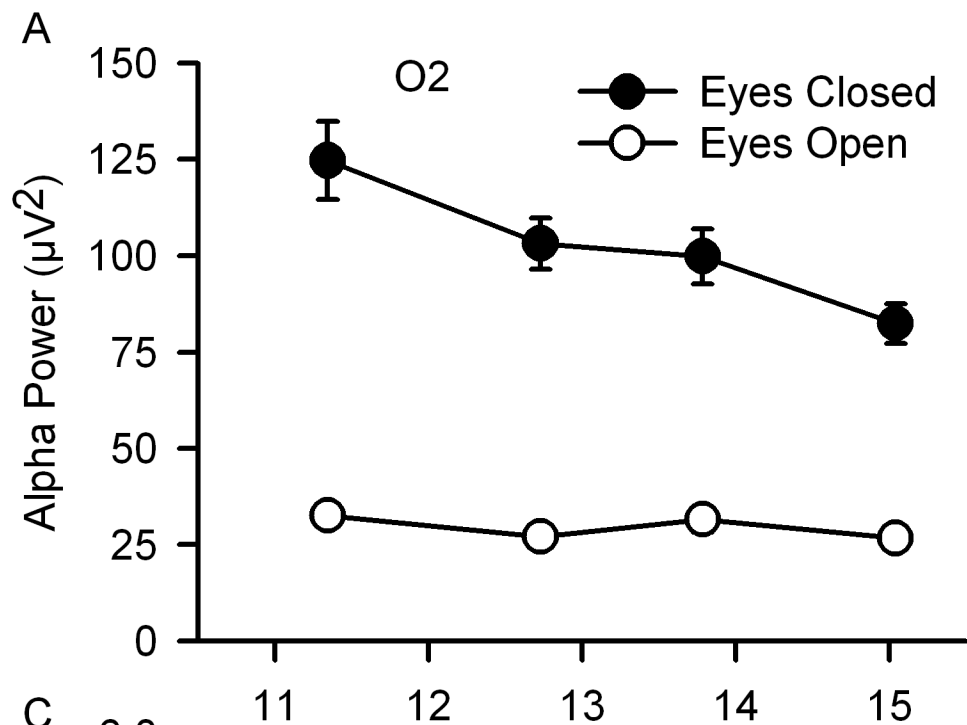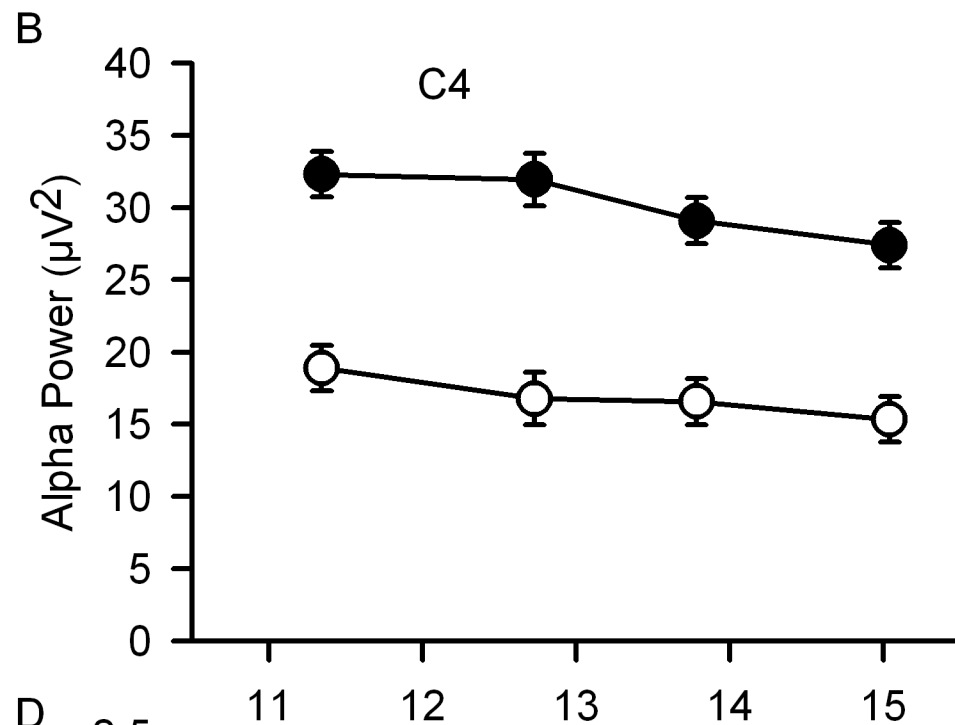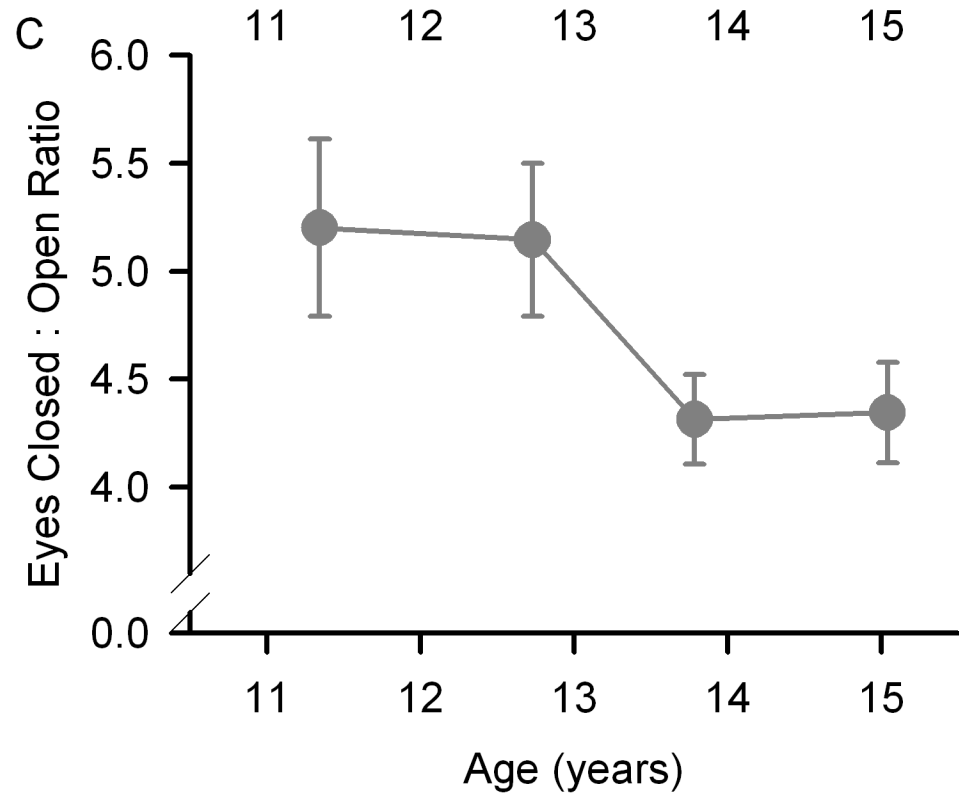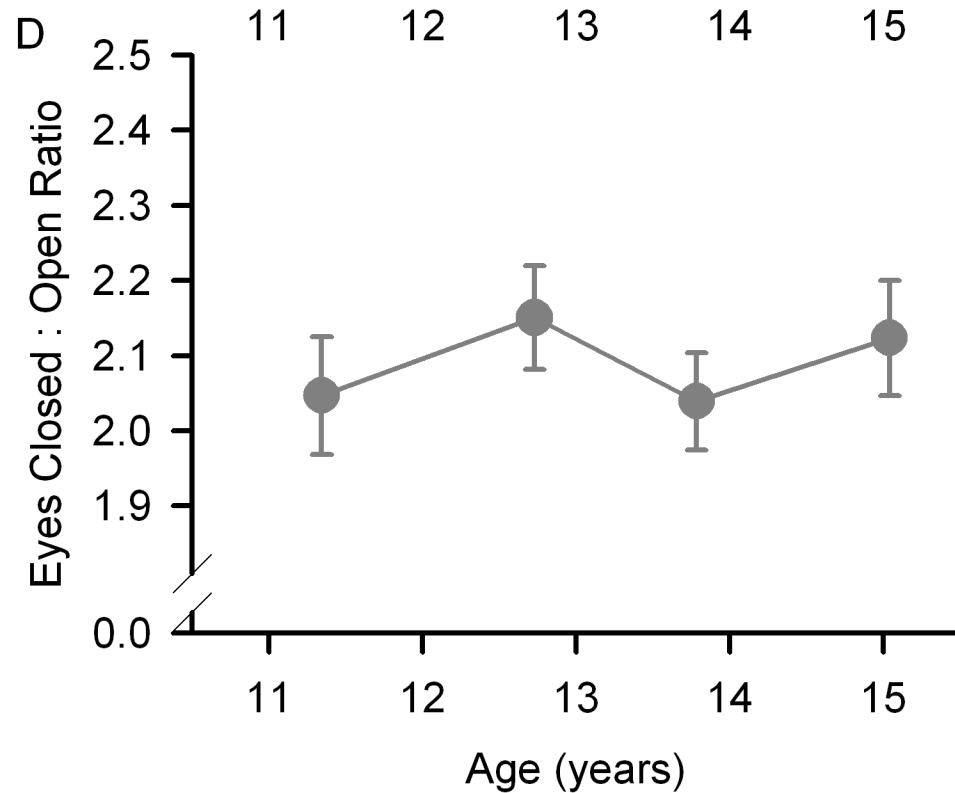

Supplement: zpac015_suppl_Supplementary_Materials [file zpac015_suppl_supplementary_materials.zip › zpac015_suppl_Supplementary_Figure_S2.PDF]

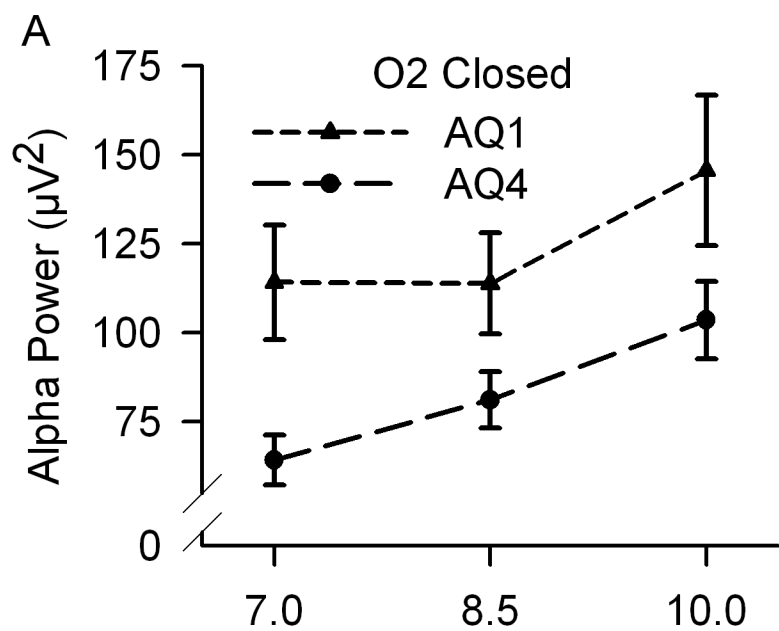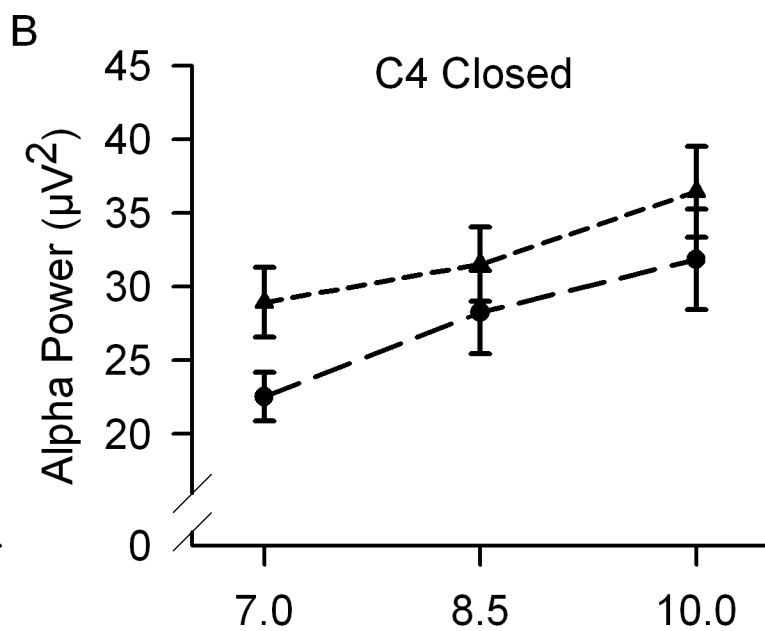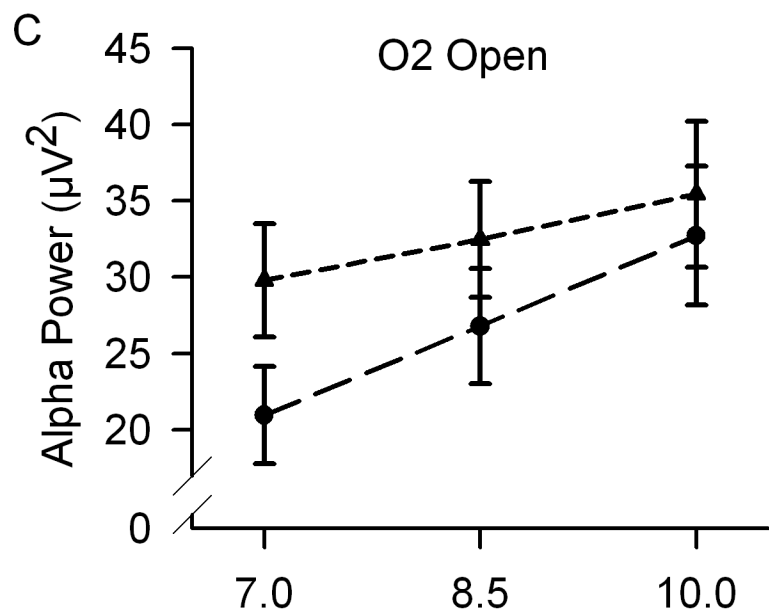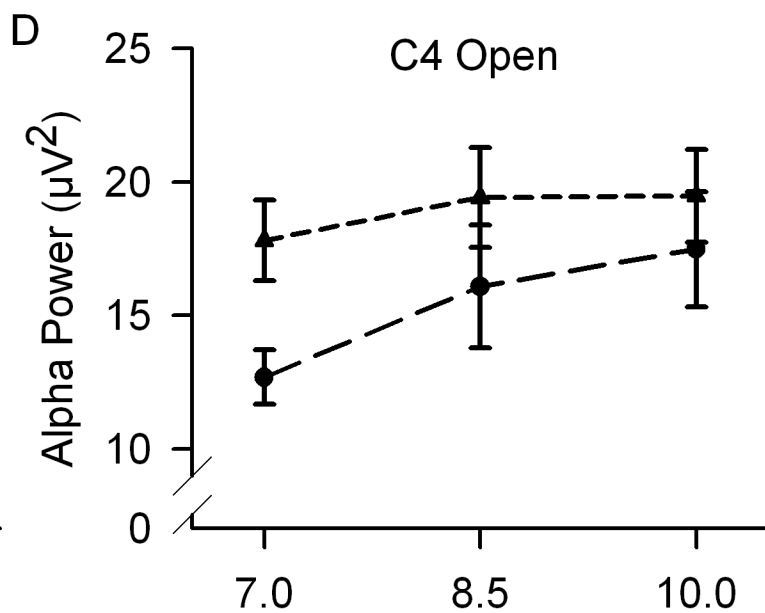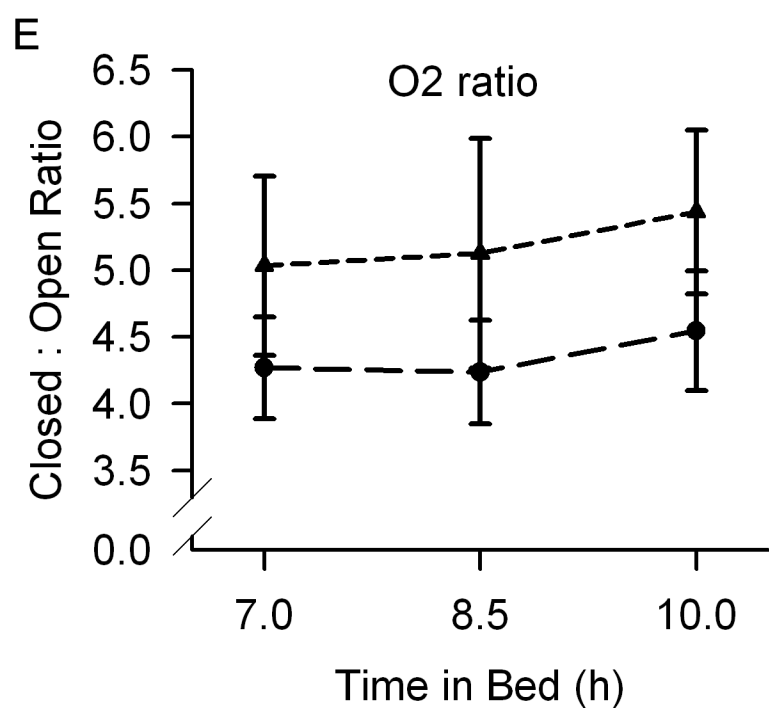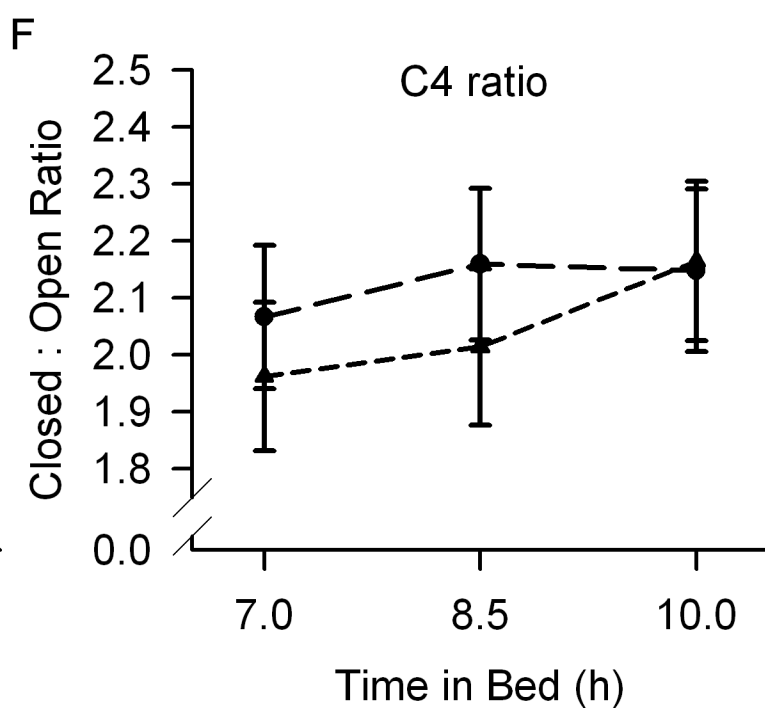

Supplement: zpac015_suppl_Supplementary_Materials [file zpac015_suppl_supplementary_materials.zip › zpac015_suppl_Supplementary_Figure_S3.PDF]

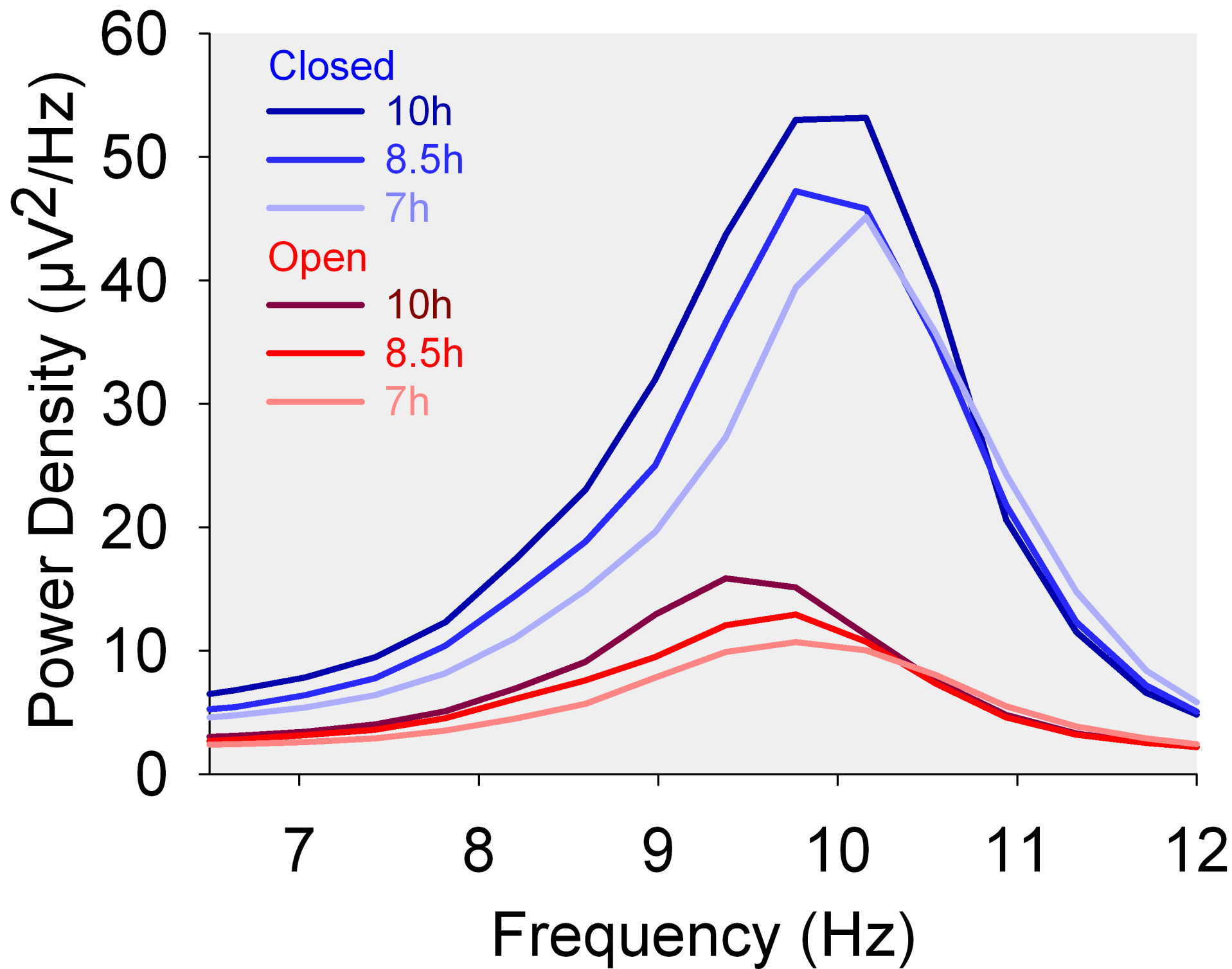

Supplement: zpac015_suppl_Supplementary_Materials [file zpac015_suppl_supplementary_materials.zip › zpac015_suppl_Supplementary_Figure_S4.PDF]

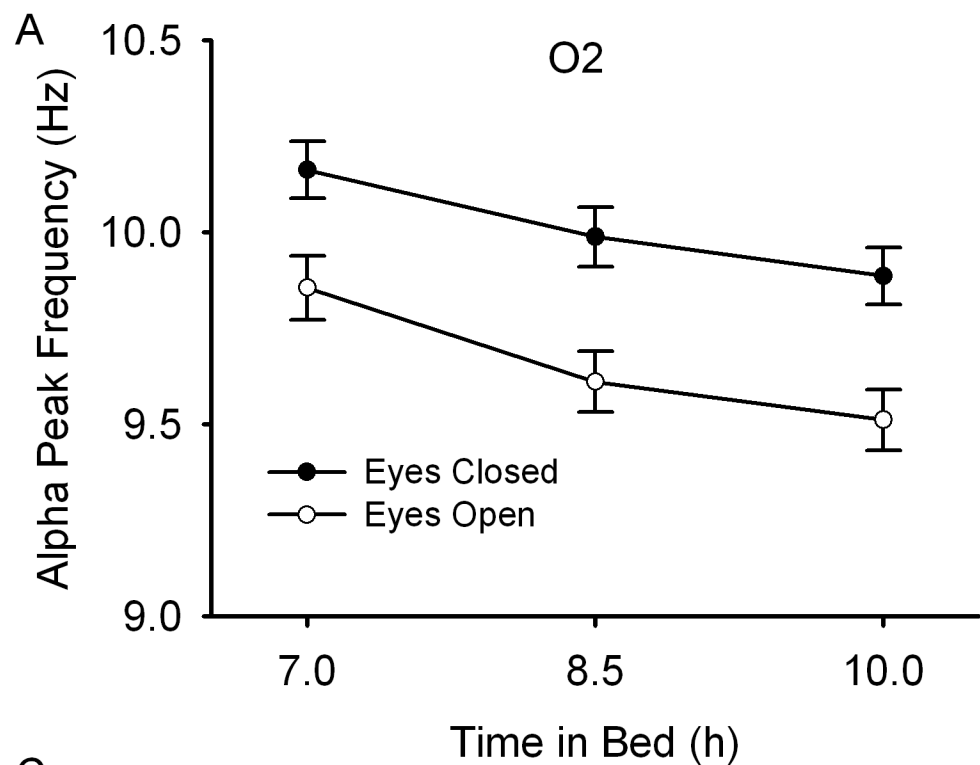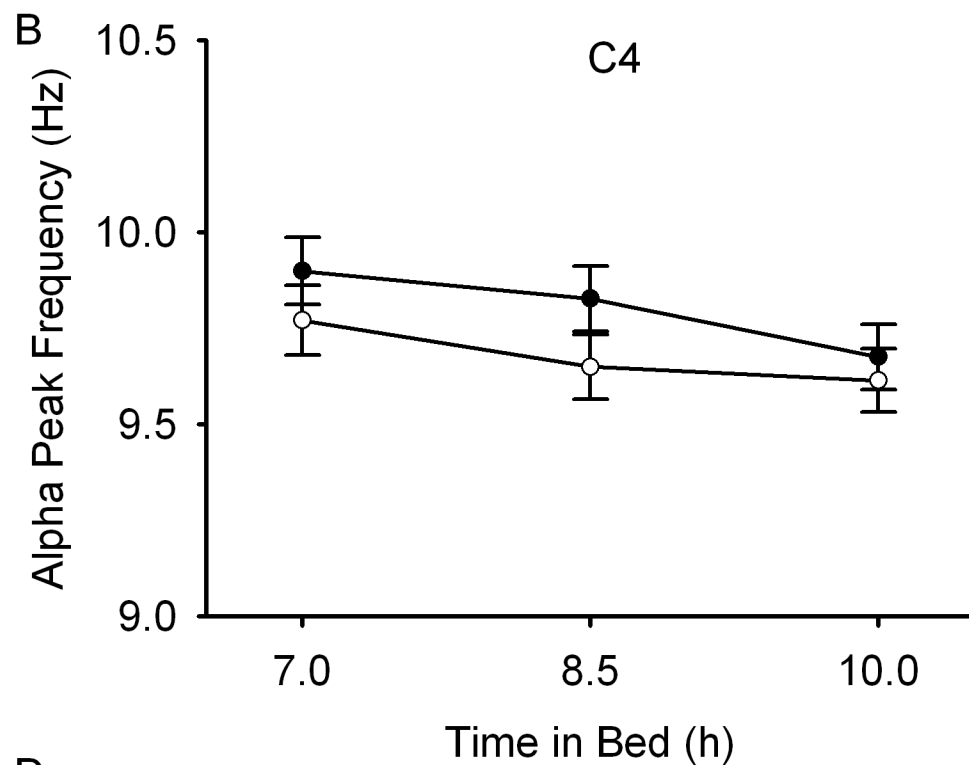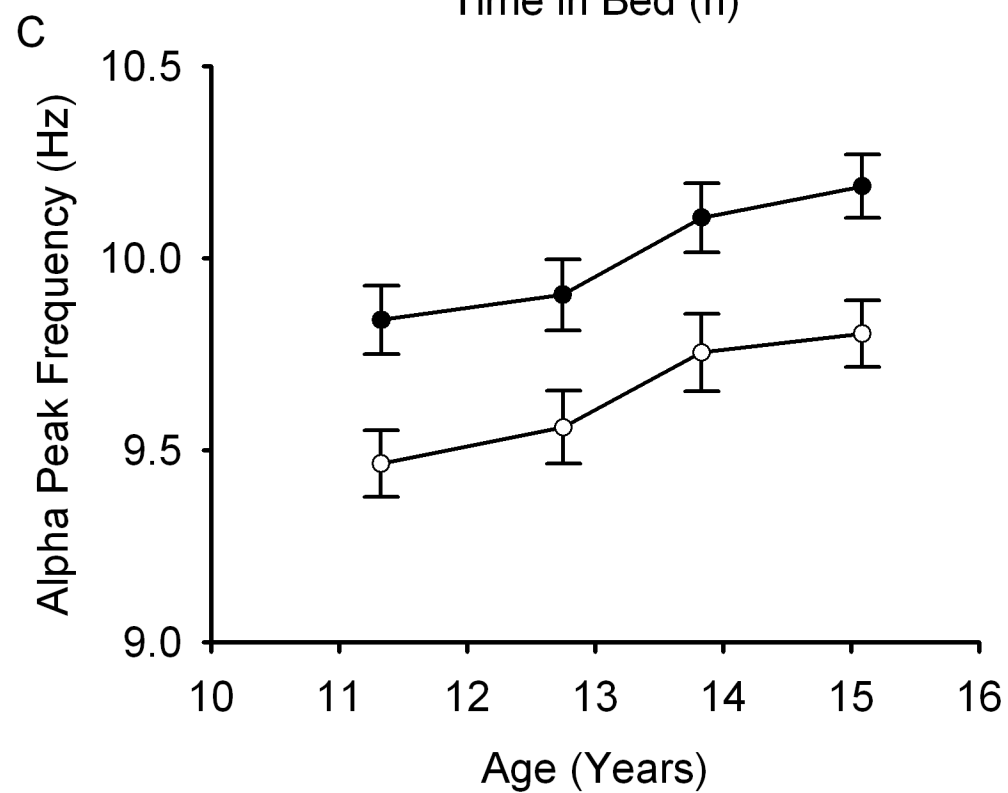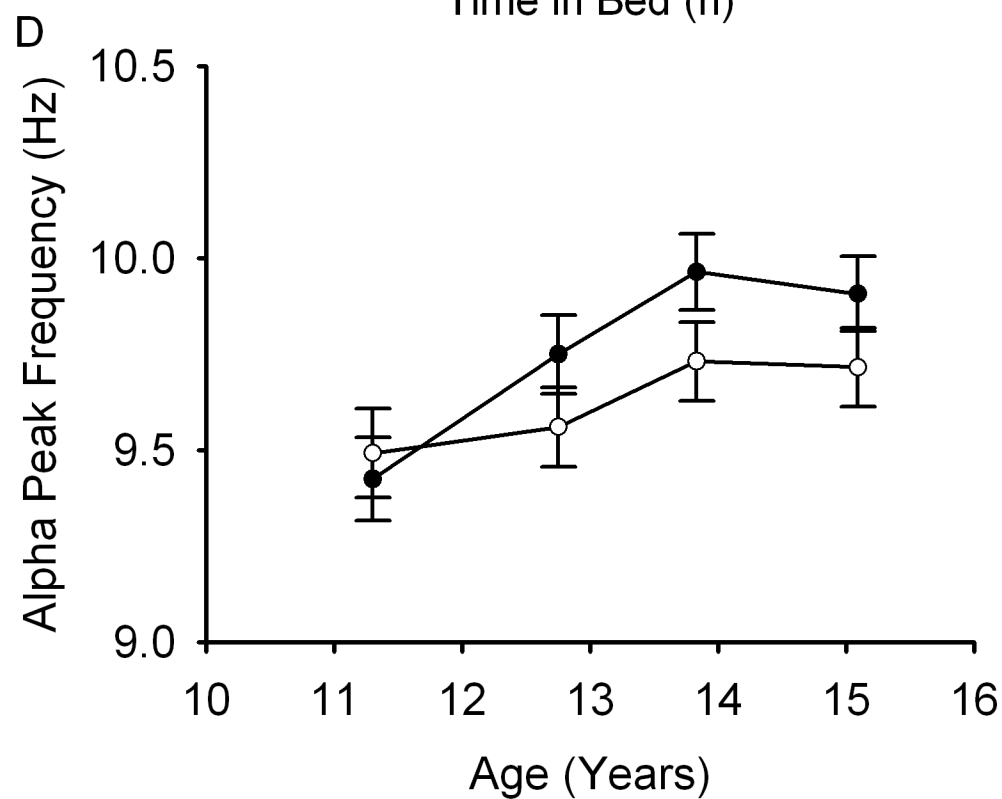

Supplement: zpac015_suppl_Supplementary_Materials [file zpac015_suppl_supplementary_materials.zip › zpac015_suppl_Supplementary_Figure_S5.PDF]
